# Etude rétrospective sur 70 cas d´hystérectomie d´hémostase dans le département de gynécologie obstétrique de l´Hôpital de Ben Arous, Tunisie

**DOI:** 10.11604/pamj.2022.42.172.34423

**Published:** 2022-07-04

**Authors:** Idriss Abidi, Hajer Bettaieb, Nesrine Souayeh, Wael Mbarki, Mohamed Frikha, Rahma Bouhmida, Hedhili Oueslati, Najeh Hsayaoui, Chaouki Mbarki

**Affiliations:** 1 Service de Gynécologie Obstétrique de l´Hôpital Régional de Ben Arous, Ben Arous, Tunisie,; 2 Université de Tunis El Manar, Faculté de Médecine de Tunis, Tunis, Tunisie

**Keywords:** Hémorragie du postpartum, hystérectomie d´hémostase, placenta accreta, inertie utérine, Postpartum haemorrhage, hemostasis hysterectomy, placenta accreta, uterine inertia

## Abstract

**Introduction:**

l´hystérectomie d´hémostase constitue le traitement radical de l´hémorragie du postpartum. Notre objectif était d´étudier les facteurs de risque, les indications et les complications de l´hystérectomie d´hémostase et de déterminer les facteurs influençant le choix du type d´hystérectomie.

**Méthodes:**

nous avons mené une étude rétrospective monocentrique descriptive et analytique dans le service de gynécologie obstétrique de l´hôpital régional de Ben Arous de 2003 jusqu´à 2019. Les patientes ont été classées selon le type d´hystérectomie totale ou subtotale.

**Résultats:**

soixante-dix patientes ont été incluses. Le taux d´hystérectomie d´hémostase était de 1,3‰. L´âge moyen était de 34,5 ans (± 5,1). Les indications d´hystérectomie d´hémostase étaient dominées par le placenta accréta dans 39% des cas (n=27), l´inertie utérine dans 34% (n=24) et la rupture utérine dans 16% des cas (n=11). Le taux de morbidité peropératoire était de 34% (n=24). Les complications les plus fréquentes étaient l´état de choc hémorragique dans 17% (n=12), la coagulation intravasculaire disséminée dans 6% (n=4) et les lésions vésicales dans 6% (n=4). Nous avons déploré six cas de décès maternel soit 8% (n=6). Une hystérectomie subtotale était réalisée chez 79% patientes (n=55) et 21% des femmes (n=15) ont eu une hystérectomie totale. Le placenta accréta était associé de manière significative au groupe hystérectomie totale (ORa: 6,93, IC 95%: 1,07-44,80, p=0,042) et la durée moyenne de l´opération était significativement plus courte en cas d´hystérectomie subtotale (ORa: 1,023, IC 95%: 1,009-1,03, p=0,01).

**Conclusion:**

l´hystérectomie est indispensable précisément dans certains cas d´hémorragie sévère du post partum. Le placenta accréta représente la principale indication d´hystérectomie. L´hystérectomie totale n´est pas associée à un risque accru de complications par rapport à l´hystérectomie subtotale.

## Abstract (en)

**Introduction:**

hemostasis hysterectomy is the radical treatment for postpartum hemorrhage. The purpose of this study is to identify risk factors, indications and complications of hemostasis hysterectomy and to determine factors influencing the types of approaches to hysterectomy.

**Methods:**

we conducted a monocentric descriptive and analytical retrospective study in the Department of Obstetrics and Gynecology at the Regional Hospital of Ben Arous from 2003 to 2019. Patients were classified according to the type of surgical treatment they received: total or subtotal hysterectomy.

**Results:**

seventy patients were included in the study. The rate of hemostasis hysterectomy was 1.3%. The average age of patients was 34.5 years (±5.1). Indications for hemostasis hysterectomy were dominated by placenta accreta (39% of cases; n=27), uterine inertia (34% of cases; n=24) and uterine rupture (16% of cases; n=11). Perioperative morbidity rate was 34 % (n=24). The most frequent complications were hemorrhagic shock (17%; n=12), disseminated intravascular coagulation (6%; n=4) and bladder lesions (6%; n=4). We reported six cases of maternal death, reflecting a rate of 8% (n=6). Subtotal hysterectomy was performed in 79% of patients (n=55) and 21% of women (n=15) underwent total hysterectomy. Placenta accreta was significantly associated with total hysterectomy group (aOR: 6.93, 95% CI: 1.07-44,80, p=0.042) and the average operation time was significantly shorter in subtotal hysterectomy group (aOR: 1.023; 95% CI: 1.009-1.03, p= 0.01).

**Conclusion:**

hysterectomy is essential in certain patients with severe postpartum hemorrhage. Placenta accreta is the main indication for hysterectomy. Total hysterectomy is not associated with an increased risk of complications compared to subtotal hysterectomy.

## Introduction

L´hémorragie du postpartum immédiat (HPPI) constitue l´une des complications les plus redoutées en obstétrique. Elle est définie par un saignement provenant du tractus génital, dépassant les 500 ml et survenant dans les 24 heures qui suivent l´accouchement [1]. Elle constitue la principale cause de mortalité maternelle, malgré les progrès en matière de prise en charge médicale, obstétricale et en radiologie interventionnelle [2,3]. L´hystérectomie d´hémostase constitue le traitement radical, elle a l´avantage d´offrir le maximum de sécurité mais au prix d´une stérilité définitive surtout pour les femmes jeunes désireuses d´autres grossesses. À ce jour, les nombreux travaux comparatifs publiés dans la littérature entre les différentes techniques chirurgicales ne permettent pas d´affirmer une supériorité statistiquement significative de l´hystérectomie totale vis-à-vis de l´hystérectomie subtotale. Au cours de ce travail, nous avons précisé les facteurs de risque, les indications, les complications, le pronostic maternel après une hystérectomie d´hémostase et déterminé les facteurs influençant le choix du type d´hystérectomie.

## Méthodes

**Conception de l´étude:** nous avons mené une étude rétrospective, monocentrique, descriptive et analytique s´étendant de janvier 2003 à décembre 2019, dans le service de gynécologie obstétrique de l´hôpital régional de Ben Arous.

**Cadre de l´étude**: il s´agit d´une maternité de niveau IIB avec une moyenne de 4500 accouchements par an.

**Population de l´étude:** il s´agissait d´une étude portant sur les patientes ayant eu une hystérectomie d´hémostase entre 2003 et 2019.

**Critères d´inclusion:** nous avons inclus toutes les patientes ayant accouché par voie basse ou par césarienne au-delà de 28 semaines d´aménorrhée, ayant présenté une hémorragie grave du postpartum dans les 24 heures suivant l´accouchement et ayant nécessité une hystérectomie d´hémostase d´emblée ou après échec du traitement chirurgical conservateur.

**Critères de non-inclusion:** les patientes non incluses étaient les femmes qui ont accouché par voie basse ou césarienne avant 28 semaines d´aménorrhée, celles qui ont présenté une hémorragie du postpartum n´ayant pas nécessité une hystérectomie et celles qui avaient eu une hystérectomie à la suite d´une hémorragie dans le postabortum ou une hémorragie d´origine autre que la sphère génitale.

**Critères d´exclusion:** nous avons exclu tous les dossiers inexploitables avec des données manquantes.

**Critères de jugements:** le critère de jugement principal était la relation entre l´indication de l´hystérectomie d´hémostase et le choix de la technique opératoire. Les critères de jugements secondaires associaient l´âge, l´utérus cicatriciel, le mode d´accouchement, les pertes sanguines, les besoins transfusionnels, le temps opératoire et les complications per et post opératoires.

**Variables étudiées:** âge, gestité, parité, antécédents médicaux et chirurgicaux, bilan prénatal, circonstances de découverte, état hémodynamique, globe, abondance du saignement, diurèse, taux d´hémoglobine, taux d´hématocrite, taux de plaquettes, taux de prothrombine, temps de céphaline activé, fibrinémie, utilisation des utérotoniques, utilisation de produits sanguins, examen sous valves, révision utérine, traitement conservateur réalisé en première intention, en deuxième intention et en troisième intention, indication de l´hystérectomie d´hémostase, durée de l´intervention, pertes sanguines, complications opératoires, rétablissement du transit, durée de l´hospitalisation, transfert en réanimation, complications post opératoires et la mortalité maternelle.

**Analyse et traitement des données:** les variables qualitatives ont été décrites en fonction de leur répartition en pourcentage avec les intervalles de confiance 95% (IC 95%). On a utilisé le test de chi deux de Pearson. Pour les variables quantitatives, l´analyse s´est faite par présentation de moyennes et écart-types quand la distribution est normale. Nous avons utilisé le test t de Student, Une analyse multivariée en régression logistique méthode descendante (Wald) a été réalisée en introduisant tous les facteurs dont les p sont < 0,05. Chaque facteur identifié a été alors présenté avec son odds-ratio ajusté (ORa) et son IC 95% Dans tous les tests, le seuil de p a été fixé à 5% (p < 0,05) et l´analyse multivariée a été réalisée à l´aide du logiciel SPSS 23.0 Fr.

**Considérations éthiques:** compte tenu de son caractère rétrospectif, le consentement n´était pas exigé. Afin de garantir la confidentialité des informations personnelles des patients, les données ont été recueillies sur des fiches d´enquête anonyme. Le protocole d´étude a été validé par le comité d´éthique de l´hôpital.

## Résultats

**Figure 1:**
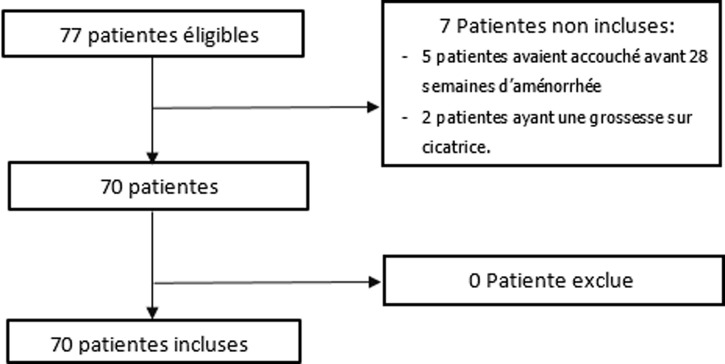
flowchart

Données épidémiologiques: durant la période d´étude, 51896 accouchements ont eu lieu. Parmi ces accouchements, nous avons recensé 70 cas d´hystérectomie d´hémostase (Figure 1) ce qui fait un taux de 1,3 ‰, soit une hystérectomie d´hémostase pour 769 accouchements. L´âge moyen était de 34,5 ans (± 5,1) [19-45]. Soixante-dix pour cent des patientes avaient un bas niveau socio-économique.

Déroulement de la grossesse: dans la majorité des cas (96%), la grossesse était mono-fœtale. Sept patientes (10%) avaient des ATCD d´hémorragie du postpartum. Dix patientes (14%) seulement avaient une grossesse bien suivie selon le programme national de périnatalité, soit 5 ou plus de consultations prénatales. Treize patientes (19%) avaient une anémie au bilan prénatal. Une hypertension artérielle gravidique a été observée chez neuf patientes (13%) et onze patientes (16%) avaient une grossesse compliquée de diabète gestationnel, un retentissement fœtal à type d´hydramnios et macrosomie a été objectivé chez six patientes (9%).

Déroulement de l´accouchement: vingt-quatre patientes (34%) avaient un travail spontané. Un déclenchement du travail a été noté chez 11 patientes (16%). Parmi ces patientes, huit (12%) ont reçu un quart de comprimé de Misoprostol et trois (4%) ont eu une sonde extra-amniotique. Un travail rapide a été noté dans 57% des cas. La majorité des patientes ont accouché par césarienne (67%). Vingt-trois patientes (33%) ont accouché par voie basse.

Données cliniques: les facteurs de risque de survenue d´HGPP étaient surtout la multiparité dans 73% des cas, l´accouchement par césarienne (67%) et l´utérus cicatriciel (60%). Le diagnostic de l´HPPI est clinique. Le maitre symptôme de l´HGPP dans notre population était le saignement observé chez 84% des patientes. Une rupture utérine a été découverte à la révision utérine dans 16% des cas.

Prise en charge médico-obstétricale: chez les patientes qui ont accouché par voie basse, l´examen sous valves et la révision utérine étaient systématiques. Chez 55 patientes (79%) nous avons eu recours à une transfusion par des culots globulaires (CGR). Le nombre de CGR médian était de 4[0-8]. Cinquante-quatre patientes (78%) ont été transfusées par du plasma frais congelé (PFC) en association avec les CGR. Le nombre de PFC médian était de 6[0-24]. Cinquante et une patientes (73%) ont eu du fibrinogène. Par manque de moyen, aucune patiente n´a reçu le facteur VII activé recombinant. L´ocytocine était administrée chez 39% des parturientes, le sulprostone (Nalador) était nécessaire chez 18 patientes (26%) toujours après échec de l´ocytocine et deux patientes (3%) ont reçu 800 milligrammes de Misoprostol par voie rectale.

Prise en charge chirurgicale: concernant le traitement chirurgical, 41 patientes (59%) ont eu une hystérectomie d´hémostase après échec du traitement conservateur et 29 patientes (41%) ont nécessité une hystérectomie d´hémostase d´emblée. Le traitement conservateur de première intention était dans la majorité des cas une ligature des artères utérines (70%). En deuxième intention, on a eu recours à une deuxième technique conservatrice chez 27 patientes (66%) soit, une triple ligature de Tsirulnikov chez 70% des cas (19/27) et une ligature bilatérale des artères hypogastriques (LBAH) chez 30% des patientes. Une hystérectomie d´hémostase a été réalisée dans un deuxième temps chez 14 patientes (34%).

**Figure 2:**
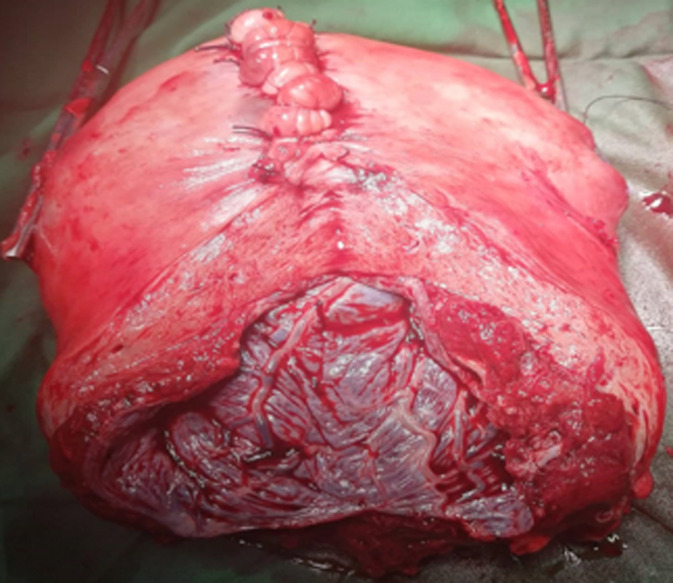
pièce d´hystérectomie en rapport avec un placenta accréta

Indications de l´hystérectomie d´hémostase: les indications d´hystérectomie d´hémostase étaient multiples et dominées par les anomalies de l´implantation placentaire en particulier le placenta accreta chez 27 patientes (39%) (Figure 2) et l´inertie utérine chez 24 patientes (34%). Les autres indications d´hystérectomie d´hémostase étaient: la rupture utérine (16%), le placenta prævia (1%), l´hématome rétro-placentaire associé à une coagulopathie (6%) et les déchirures cervicales complexes (3%). Une hystérectomie d´hémostase a été faite suite à l´impossibilité de suturer les berges d´un fibrome prævia avec hémorragie de grande abondance chez une patiente. La durée moyenne de l´intervention chirurgicale était de 180,86 minutes [± 8,34]. Le taux de morbidité peropératoire était de 34%. Les complications les plus fréquentes étaient, par ordre de fréquence, l´état de choc hémorragique (17%), la CIVD (6%) et les lésions vésicales (6%). La Delta hémoglobine moyenne était de 2,54 g/dl. Onze patientes (16%) ont été transférées en réanimation. Les complications postopératoires étaient observées chez 11 patientes soit 16% des cas. Ces complications étaient dominées par l´insuffisance rénale aigue et l´état de choc hémorragique. Aucun accident transfusionnel n´a été noté. La durée médiane de l´hospitalisation était de sept jours [3-30]. Nous avons déploré six cas de décès maternel (8%). Trois décès étaient survenus en peropératoire et trois décès étaient recensées en postopératoire (Tableau 1). Une hystérectomie subtotale (HST) était réalisée chez 55 patientes (79%). Le reste ont eu une hystérectomie totale (HT). Il n´y avait pas de différence statistiquement significative dans l´incidence des complications entre les groupes HST et HT. La comparaison des données des patientes par l´analyse multivariée par régression logistique a permis de retenir le placenta accreta et la durée d´intervention comme facteurs influençant le choix du type de l´hystérectomie. Ainsi le placenta accreta était associé de manière significative au groupe HT (ORa:6,93, IC 95%: 1,07-44,80, p=0,042) et la durée moyenne de l´opération était significativement plus courte pour le groupe HST que pour le groupe HT (ORa: 1,023, IC 95%: 1,009-1,03, p=0,01) (Tableau 2).

**Tableau 1:** caractéristiques des différentes patientes décédées durant l´étude

Cas	Âge	ATCD	Voie d´accouchement	Indication de l´hystérectomie	Cause du décès	Heure du décès
1	31	G2P2, utérus cicatriciel	AVB	Inértie utérine	CIVD	J1 Post-opératoire
2	39	G4P4	AVB	Inértie utérine	CIVD	J1 Post-opératoire
3	19	G1P1	Cs	Inértie utérine	EDC hémorragique +CIVD	J3 Post-opératoire
4	29	G2P2	Forceps	Déchirure cervicale complexe	EDC hémorragique	En pér-opératoire
5	36	G6P4	AVB	Rupture utérine	EDC hémorragique +CIVD	En pér-opératoire
6	34	G3P3, bicicatriciel	Cs	Placenta accreta	EDC hemorragique +Hypokaliémie	En pér-opératoire

**ATCD**: antécédents ;**EDC**: état de choc; **CIVD**: coagulation intravasculaire disséminée; **AVB**: accouchement voie basse ; Cs: césarienne

**Tableau 2:** facteurs influençant le choix du type d´hystérectomie totale ou subtotale

Variables	OR	ρ	IC95%	ORa	ρ	IC95%
Age		0,14	-3.87- 0.52			
Parité		0,15				
Terme d´accouchement		0,12				
Utérus cicatriciel	5,8	**0,02**	1,19- 28,27	2,597	0,388	0,26-22,68
Placenta accreta	8,05	**0,002**	2,2-29,41	**6,93**	**0,042**	1,07-44,80
Inertie utérine	0,09	**0,013**	0,01-0,81	0,229	0,267	0.17-3,08
Rupture utérine	0,32	0,435	0,03-2,75			
HRP avec coagulopathie	0,77	0,571	0,67-0,88			
Placenta praevia	0,78	0,99	0,69-0,88			
Nombre médian en CG		0,246				
Nombre médian en PFC		0,536				
Lésions vésicales		0,571				
CIVD	0,76	0,329	0,66-0,87			
Décès	0,76	0,329	0,66-0,87			
Durée d´intervention		**<0,002**		**1,023**	**0,01**	1,009-1,03

ρ: degré de significativité; OR: odds-ratio; ORa: odds-ratio ajusté; HRP: hématome retro-placentaire; CIVD: coagulation intravasculaire disséminée, IC95%: intervalle de confiance

## Discussion

Dans notre travail, 70 patientes ont été incluses. L´analyse a montré que le placenta accreta était l´indication la plus fréquente de l´HH. Quarante et une patientes ont eu une HH après échec du traitement conservateur et 29 patientes ont nécessité une HH d´emblée. Une HST était réalisée chez 55 patientes. Les facteurs influençant le choix du type de l´hystérectomie d´hémostase étaient le placenta accréta et la durée d´intervention. La fréquence de l´HH reste encore élevée dans le monde malgré les progrès réalisés dans la prise en charge de l´HGPP, avec une prédominance dans les pays du sud [4,5]. Durant la période de notre étude, nous avons recensé un taux de 0,13%. Cette fréquence est inférieure à celle rapportée dans les pays sous-développés mais encore élevée par rapport aux pays développés [6-9].

L´étude italienne de Barillari *et al*. a décrit un âge moyen de 34,5 ans [10]. Les résultats de notre étude étaient concordants à ceux avancés par la littérature. Le niveau socio-économique prend toute sa valeur dans les pays en voie de développement où un faible niveau socio-économique est associé à une précarité du système de santé, à une carence nutritionnelle et à l´anémie influençant négativement l´incidence des HGPP [5]. Selon les données épidémiologiques rapportées par Ahmadi *et al*. [11], 75% des parturientes avaient un niveau socio-économique précaire. Dans notre série, 70% des parturientes étaient issues d´un milieu socio-économique défavorable. Zelop *et al*. [12] ont démontré que l´incidence de l´HH augmente de façon significative avec la parité. De même Zelop *et al*. ont montré que la multiparité est un facteur de risque d´HH [12]. En effet la multiparité est un facteur de fragilisation de l´utérus favorisant la rupture utérine, l´inertie utérine et l´inversion utérine ainsi que l´hémorragie du postpartum [13]. Ces résultats sont similaires à ceux retrouvés dans notre travail, où 63% des patientes étaient des multipares. Par ailleurs, Combs *et al*. [14] ont constaté que la primiparité est un facteur de risque de prééclampsie, de travail anormal, de lésion de la filière génitale pouvant ainsi être responsable aussi de l´hémorragie du postpartum. Quatre pour cent de nos patientes étaient primipares, alors que dans la littérature ce taux est disparate [15,16]. Selon les données de la littérature, l´utérus cicatriciel est un facteur de risque de l´HH [12,17-21]. En outre, l´utérus cicatriciel augmente le risque d´anomalies d´adhésion et d´insertion placentaire majorant ainsi le risque de survenue d´une hémorragie de la délivrance et d´HH. En effet, l´incidence du placenta accréta semble être augmentée et cette augmentation est proportionnelle au nombre de césariennes antérieures chez les patientes présentant un placenta prævia. L´étude de Clark *et al*. a décrit que le risque de placenta accréta était de 24% en cas d´un utérus unicicatriciel, et atteint 67% en cas d´utérus quadricicatriciel [20].

Notre étude est venue également réconforter ces résultats, l´antécédent d´utérus cicatriciel était retrouvé chez 60% des patientes. L´existence d´antécédents d´HGPP est un des facteurs de risque les plus associés, mais reste peu prévalent. Ford *et al*. ont rapporté que le risque d´HGPP est multiplié par trois en cas d´antécédent d´HPP [22]. Les études récentes conduisent à penser que les consultations prénatales de suivi des grossesses permettent de mettre en place des interventions d´efficacité prouvée pour la mère et le nouveau-né. Ces consultations ont un rôle fondamental dans la réduction de la morbidité et la mortalité maternelle et l´absence d´un suivi prénatal est un facteur identifié d´hystérectomie d´hémostase [23]. D´autant plus que dans notre étude seulement dix patientes (14%) avaient une grossesse bien suivie selon le programme national de périnatalité. Nwobodo *et al*. ont rapporté que la survenue des événements indésirables comme l´HH était statistiquement liée au suivi prénatal, notant une incidence de 1,82% chez les patientes n´ayant pas eu de suivi prénatal comparée à 0,07% chez les patientes suivies [5]. Dans l´étude de Nyflot *et al*., l´anémie au cours de la grossesse était considérée comme facteur de risque d´HH. Ceci peut être expliqué par la baisse du seuil de tolérance à des pertes sanguines même moyennes en cas d´anémie [24]. Plusieurs pathologies gravidiques telles que la pré-éclampsie, la mort fœtale in utero (MFIU), la stéatose hépatique aigue gravidique (SHAG) et l´hématome rétro-placentaire (HRP) constituent des situations favorisantes d´un trouble acquis de l´hémostase. Combs et Reyal considéraient que la toxémie gravidique est l´un des principaux facteurs de risque d´HH avec des OR respectifs de 5,02 et 6,92 [14,25]. Des études expérimentales ont prouvé qu´une exposition prolongée à l´ocytocine pendant le travail pouvait entrainer la désensibilisation et la saturation de ses récepteurs. Ceci entraine une interruption de la cascade de signalisation intracellulaire limitant la contraction médiée par l´ocytocine [14,26-28]. Dans notre série, une direction de travail a été faite chez 21% des patientes. La transfusion fait l´objet de recommandations générales. Différentes études concluent que la transfusion de produits sanguin labiles (PSL) est un élément primordial dans la prise en charge des HPPI [29,30].

Les indications d´HH sont multiples. La littérature rapporte que les anomalies d´insertion placentaire est actuellement le premier motif de réalisation d´une HH [31,32]. L´étude récente de Flood *et al*. a étudié le changement des indications de l´HH en montrant une augmentation significative des anomalies d´adhésion placentaire de 5,4% à 46,5% (p <0,001) et une réduction significative du taux de l´atonie utérine de 40,5% à 9,3% (p <0,001) [33]. L´incidence du placenta accréta a été multipliée par dix dans les cinquante dernières années et serait de 1/2500 accouchements [34]. Cette élévation est expliquée par l´augmentation constante du taux de césarienne dans le monde et elle est proportionnelle au nombre de césariennes antérieures chez les patientes ayant un placenta prævia [34]. En raison du fort taux d´échec des mesures conservatrices, la césarienne-hystérectomie en un temps est le traitement de référence du placenta accréta selon le Collège Américain de Gynécologie Obstétrique [35,36]. Le diagnostic anténatal du placenta accréta est capital. Il doit être réalisé chez toute femme présentant un ou plusieurs facteurs de risque. Il se base essentiellement sur l´échographie couplée au doppler et l´imagerie par résonnance magnétique en cas de placenta postérieur [37]. En effet, Warshak *et al*. ont montré que les patientes dont le placenta accréta était diagnostiqué en anténatal et chez qui une césarienne hystérectomie sans tentative de délivrance artificielle était alors programmée à 34-35 SA, recevaient moins de culots globulaires et avaient tendance à moins saigner en comparaison avec les patientes dont le placenta accréta n´avait pas été diagnostiqué [38]. En termes de fréquence, la deuxième indication d´hystérectomie d´hémostase dans notre étude était l´atonie utérine avec 34%. L´étude de Habek *et al*. a montré que le taux d´HH due à une inertie utérine était de 25% [9]. La diminution de l´incidence de l´inerte utérine rapportée par plusieurs auteurs est expliquée par l´efficacité du traitement médical (les utérotoniques et les prostaglandines) et le développement des techniques chirurgicales conservatrices [33]. Dans notre série, le taux de rupture utérine était de 16%. Ce taux rejoint celui de Rahman *et al*. qui était de 18% [39]. Flood *et al*. ont décrit une diminution significative au cours de cette décennie de cette indication de 40% à 9% [33]. Cette baisse significative est probablement le résultat de l´évolution de la pratique obstétricale, la diminution de la parité des patientes et l´utilisation plus judicieuse de l´ocytocine.

Dans notre travail, une HST était réalisée chez 79% des patientes. Smith *et al*. n´ont pas trouvé une différence significative concernant le temps opératoire, les indications et les complications per et postopératoires entre les deux groupes HST et HT [40]. Zhang *et al*. ont trouvé que la durée moyenne de l´opération était significativement plus courte pour le groupe HST par rapport au groupe HT et que les besoins transfusionnels en CGR étaient plus importants dans le groupe HT [41]. Cependant, Flood *et al*. ont montré que l´HT était significativement plus efficace en matière de contrôle de saignement d´origine cervicale [33]. Les résultats contradictoires de la littérature peuvent être expliqués par la randomisation extrêmement difficile à envisager dans un contexte d´urgence vitale, mais aussi la stratégie globale de prise en charge et en particulier les mesures médicales, obstétricales et radiologiques variant en fonction des équipes, du plateau technique et des moyens humains. Les patientes et les indications sont également très différentes, notamment en ce qui concerne la gravité de l´hémorragie au moment où est décidée l´intervention chirurgicale. Les études ont de ce fait des références de niveau de preuve faible et les stratégies proposées reposent le plus souvent sur un consensus professionnel obtenu au sein d´un groupe de travail ou d´une équipe [37]. Il semble en revanche plus intéressant de considérer que c´est l´indication même de l´hystérectomie qui guide l´opérateur en fonction de son appréciation clinique et de son expérience. L´indication d´une hystérectomie totale est indispensable en cas d´hémorragie d´origine segmentaire inférieure ou cervicale qui se voit surtout dans le placenta prævia accréta et les déchirures cervicales complexes. Dans les autres cas, et notamment en cas d´atonie utérine, l´hystérectomie subtotale apparaît comme une alternative acceptable [37]. En peropératoire, les plaies des organes avoisinants sont des accidents rencontrés au cours de l´hystérectomie d´hémostase. Selon Harris, les complications les plus fréquentes sont les plaies vésicales suivies des plaies intestinales [42]. Dans la littérature, des lésions vésicales et urétérales en peropératoire ont été retrouvées dans 4 à 16% [43,44]. Les lésions vésicales étaient plus élevées de manière significative chez les femmes présentant un placenta accréta que chez les femmes présentant une atonie utérine [45]. Pradhan *et al*. ont montré que les complications peropératoires étaient dominées par la CIVD dans 37,7% des cas suivies de l´état de choc hémorragique dans 26,2% des cas [43]. Dans notre travail, 6% des patientes ont présenté une insuffisance rénale aiguë nécessitant des séances d´hémodialyse. Cette insuffisance rénale est liée à une hypo perfusion des reins faisant suite à la spoliation sanguine. Ces résultats sont similaires à ceux trouvés dans la série de Ducarme *et al* ou le taux d´insuffisance rénale était de 6.2% [46]. Dans les pays développés, le taux de la mortalité après HH est faible [47-49]. Alors que dans les pays en voie de développement, ce taux est encore élevé [16,50]. Dans notre étude, nous avons enregistré six décès maternels soit un taux de 8%. Les causes de décès maternel les plus fréquentes, dans notre série, étaient la CIVD et l´état de choc hémorragique. Ces résultats sont en concordance avec les constatations décrites par Zeteroglu *et al*. et Chawla *et al*. [16,51]. Notre étude présente les points forts suivants: la population étudiée dans notre travail est considérée comme un échantillon assez représentatif diminuant le biais de sélection avec un recul suffisant pour évaluer les résultats à long terme. Néanmoins, elle présente certaines limites qui sont surtout d´ordre méthodologique liées notamment au caractère rétrospectif et monocentrique de l´étude.

## Conclusion

Le taux d´HH dans notre série reste élevé par rapport aux pays développés. Les facteurs de risque d´hystérectomie d´hémostase les plus fréquents étaient la multiparité, l´accouchement par césarienne, et l´utérus cicatriciel. Le placenta accréta représente la principale indication d´hystérectomie dans notre étude. Bien que le temps opératoire fût plus long, l´hystérectomie totale n´était pas associée à un risque accru de complications par rapport à l´hystérectomie subtotale. Seulement 10% de nos patientes avaient un bon suivi de leurs grossesses. Ainsi, nous insistons sur l´intérêt du suivi prénatal.

### Etat des connaissances sur le sujet

- L´hystérectomie est indispensable précisément dans certains cas d´hémorragie sévère du post partum, malgré les progrès en matière de prise en charge médicale, obstétricale et en radiologie interventionnelle de l´hémorragie grave du postpartum;
- Les données publiées dans la littérature entre l´hystérectomie totale et subtotale ne permettent pas d´affirmer une supériorité statistiquement significative d´une technique par rapport à l´autre.

### Contribution de notre étude à la connaissance

- La fréquence de l´hystérectomie d´hémostase est encore élevée par rapport aux pays développés;
- Le placenta accréta représente la principale indication d´hystérectomie;
- Bien que le temps opératoire soit plus long, l´hystérectomie totale n´est pas associée à un risque accru de complications par rapport à l´hystérectomie subtotale.
